# Elevated Hexose-6-Phosphate Dehydrogenase Regulated by OSMR-AS1/hsa-miR-516b-5p Axis Correlates with Poor Prognosis and Dendritic Cells Infiltration of Glioblastoma

**DOI:** 10.3390/brainsci12081012

**Published:** 2022-07-30

**Authors:** Yi-Bin Zhang, Shu-Fa Zheng, Lin-Jie Ma, Peng Lin, Huang-Cheng Shang-Guan, Yuan-Xiang Lin, De-Zhi Kang, Pei-Sen Yao

**Affiliations:** 1 Department of Neurosurgery, Neurosurgical Research Institute, First Affiliated Hospital, Fujian Medical University, Fuzhou 350004, China; eabin.z@fjmu.edu.cn (Y.-B.Z.); zsf2002110@163.com (S.-F.Z.); sghc8798@163.com (H.-C.S.-G.); lyx99070@163.com (Y.-X.L.); 2 Department of Neurology and Neurosurgery, Changji Traditional Chinese Medicine Hospital, Changji 831100, China; linjiema110@163.com; 3 Department of Pain, First Affiliated Hospital, Fujian Medical University, Fuzhou 350004, China; 909038896@163.com; 4 Fujian Key Laboratory of Precision Medicine for Cancer, First Affiliated Hospital, Fujian Medical University, Fuzhou 350005, China; 5 Key Laboratory of Radiation Biology of Fujian Higher Education Institutions, First Affiliated Hospital, Fujian Medical University, Fuzhou 350005, China; 6 Fujian Provincial Institutes of Brain Disorders and Brain Sciences, First Affiliated Hospital, Fujian Medical University, Fuzhou 350005, China

**Keywords:** H6PD, ceRNA network, glioblastoma, immune infiltration, outcome

## Abstract

Objective Glioblastoma (GBM), a type of malignant glioma, is the most aggressive type of brain tumor and is associated with high mortality. Hexose-6-phosphate dehydrogenase (H6PD) has been detected in multiple tumors and is involved in tumor initiation and progression. However, the specific role and mechanism of H6PD in GBM remain unclear. Methods We performed pan-cancer analysis of expression and prognosis of H6PD in GBM using the Genotype-Tissue Expression Project (GTEx) and The Cancer Genome Atlas (TCGA). Subsequently, noncoding RNAs regulating H6PD expression were obtained by comprehensive analysis, including gene expression, prognosis, correlation, and immune infiltration. Finally, tumor immune infiltrates related to H6PD and survival were performed. Results Higher expression of H6PD was statistically significantly associated with an unfavorable outcome in GBM. Downregulation of hsa-miR-124-3p and hsa-miR-516b-5p in GBM was detected from GSE90603. Subsequently, OSMR-AS1 was observed in the regulation of H6PD via hsa-miR-516b-5p. Moreover, higher H6PD expression significantly correlated with immune infiltration of dendritic cells, immune checkpoint expression, and biomarkers of dendritic cells. Conclusions The OSMR-AS1/ miR-516b-5p axis was identified as the highest-potential upstream ncRNA-related pathway of H6PD in GBM. Furthermore, the present findings demonstrated that H6PD blockading might possess antitumor roles via regulating dendritic cell infiltration and immune checkpoint expression.

## 1. Introduction

Glioma tissue derived from the nerve epithelium is a diffuse, highly invasive cerebral tumor with a poor prognosis. Glioblastoma (GBM), a type of malignant glioma, is the most aggressive variety of brain tumor and is associated with high mortality. GBM patients typically have short survival times (approximately 1 year) and an extremely poor prognosis, with a 5-year overall survival (OS) rate of less than 4% [1]. The prognosis of GBM patients remains dismal despite multiple therapies such as radical surgery, chemotherapy, and radiotherapy [2,3]. Thus, detailed underlying molecular mechanisms and prognostic predictors in GBM patients are urgently needed.

Hexose-6-phosphate dehydrogenase (H6PD) is involved in the downregulation of nicotinamide adenine dinucleotide phosphate (NADPH) in the endoplasmic reticulum lumen via converting glucose 6-phosphate to 6-phosphogluconate [4,5]. The H6PD gene is upregulated in multiple malignancies, and early studies reported that H6PD led to cancer cell growth [6,7]. Knockdown of H6PD reduced the proliferation and migration of breast cancer cells [5]. Downregulation of H6PD with short interfering RNAs in the murine colon and breast cancer cells correlated with reduced glucose uptake and decreased proliferation [8]. Silencing of H6PD contributed to reduced invasion and migration of gallbladder cancers [7]. Furthermore, increased H6PD plays a crucial role in purine metabolic reprogramming and the progression of glioma stem cells [9].

Nevertheless, the prognosis and regulatory mechanisms of H6PD in GBM remain scarcely explored. Moreover, the potential function of H6PD in regulating tumor immune cell infiltration in GBM is incompletely understood. In this study, we performed survival analysis for H6PD in a pan-cancer study. Next, the potential upstream (noncoding RNA) pathways of H6PD, including long noncoding RNAs (lncRNAs) and microRNAs (miRNAs), are also explored in GBM. Finally, we explored the correlation between H6PD expression and immune infiltration in GBM. Collectively, our study suggests that elevated H6PD regulated by OSMR-AS1/hsa-miR-516b-5p correlates with tumor immune infiltration and poor prognosis in GBM patients.

## 2. Materials and Methods

### 2.1. TCGA Data Download, Process, and Analysis

Pan-cancer gene expression data were acquired from The Cancer Genome Atlas (TCGA) database (https://tcga-data.nci.nih.gov/tcga/, accessed on 15 February 2022). We analyzed the differential expression of H6PD using the R package [10]. We also analyzed weighted Pearson correlations and *p*-values using the R package “weights” (https://CRAN.R-project.org/package=weights); *p*-values less than 0.05 were considered statistically significant.

### 2.2. GEPIA Database Analysis

GEPIA (http://gepia.cancer-pku.cn/detail.php, accessed on 15 February 2022) is a web-based tool for gene expression and correlation analysis based on TCGA and data from the Genotype-Tissue Expression Project (GTEx) [11]. GEPIA was used to analyze the expression of H6PD and long noncoding RNA (lncRNA) in different types of cancers. Survival analysis for H6PD in pan-cancer studies, including overall survival (OS) and disease-free survival (DFS), was conducted using GEPIA. In addition, prognostic values of the candidate lncRNAs were assessed by GEPIA and the R package. PDCD1, CD274, CTLA4, SIGLEC15, HAVCR2, TIGIT, LAG3, and PDCD1LG2 were selected as major immune checkpoints. The associations between H6PD and immune checkpoints in GBM were investigated using data from the GEPIA database. 

### 2.3. ENCORI Database Analysis

ENCORI (http://starbase.sysu.edu.cn/, accessed on 17 February 2022) is a web server for analyzing RNA-related studies [12]. Candidate miRNAs were obtained using ENCORI. Several target prediction programs obtained predictive miRNAs of H6PD, including RNA22, miRmap, PITA, microT, miRanda, PicTar, and TargetScan. In addition, parameters for degradome data (low stringency), clip data (medium stringency), and pan-cancer type (one cancer type) were set. Only predicted miRNAs acquired in at least three programs were considered candidate miRNAs of H6PD. The correlation between miRNAs and H6PD in GBM was analyzed by using ENCORI. miRNAs negatively correlated with H6PD were selected for subsequent survival analysis. In addition, candidate lncRNAs that might bind to candidate miRNAs were obtained from ENCORI.

### 2.4. Differential Expression Analysis by microRNA-seq

The miRNA sequencing of glioblastoma is publicly available from the GEO under accession number GSE90603. The reads were mapped to the human genome (hg38). The resulting matrix of reading counts was then loaded into RStudio (R version 3.6.3, R core Team, 2020, R Foundation for Statistical Computing, Vienna, Austria). The differential expression analysis was performed using the R/Bioconductor package DESeq (version 1.16.0, R Foundation for Statistical Computing, Vienna, Austria). 

### 2.5. GEO2R Analysis

GEO2R (http://www.ncbi.nlm.nih.gov/geo/geo2r, accessed on 21 February 2022), a web tool available from the NCBI, was applied to identify differentially expressed genes between GBM and normal samples. GEO2R carried out comparisons using the GEOquery and Limma R packages from the Bioconductor project to identify the differentially expressed miRNAs in GBM and normal brain samples. The statistical significance of miRNA differential expression was determined by assigning |Log2 fold change| (|log2FC|) values and adjusted *p*-values. An adjusted *p*-value < 0.05 and the absolute|log2FC| > 1 were set as the cut-off criteria.

### 2.6. Prediction of lncRNA and ceRNA Network Construction

Based on the ceRNA hypothesis [13,14], we analyzed the positive correlation between H6PD and targeted lncRNAs. We constructed a lncRNA–miRNA–mRNA interaction network for H6PD to understand the gene regulation of H6PD. 

### 2.7. UCSC Xena Database and Kaplan-Meier Plotter Analysis

The UCSC Xena database (http://xena.ucsc.edu/, accessed on 22 February 2022) is a visualization and analysis web tool for correlations between genomic and phenotypic variables. The database contains TCGA data. The database provided information on survival outcomes and gene expression. The expression and survival curves of lncRNAs were acquired by combining the GEPIA database and the “survival” package-derived R Project. 

### 2.8. TIMER Database Analysis

TIMER (https://cistrome.shinyapps.io/timer/, accessed on 21 February 2022) is a classical and authoritative database for evaluating immune infiltrates and clinical implications in diverse cancer types. TIMER was used to generate the correlation of H6PD expression with immune infiltrates in GBM. The survival module investigated the association between clinical outcomes and immune infiltrates.

### 2.9. Immune Infiltration Analysis

Tumor immune infiltrates were obtained using a single GSEA (ssGSEA) method with a GSVA R package based on TCGA datasets [15]. The correlation between H6PD and eight immune cell types was determined using the Spearman correlation test. Graphs were obtained using the ggplot2 R package (version 3.6.3, R core Team, 2020, Vienna, Austria). The GEPIA database was utilized to identify the relationships between H6PD and biomarkers of immune cells.

### 2.10. UACLAN 

UALCAN is designed to provide easy access to a publicly available database; it is a comprehensive and interactive online tool that can be used to analyze cancer OMICS data [16]. Graphs and plots describing survival information of lncRNAs in GBM patients were obtained from UALCAN. 

### 2.11. The Human Protein Atlas (THPA) Analysis

THPA was used to assess the H6PD expression of GBM and normal brain tissues based on immunohistochemistry. 

### 2.12. Statistical Analysis

H6PD expression analysis was performed with TIMER, GEPIA, THPA, and R projects using the “ggplot2” package. Survival analysis was performed with GEPIA, TIMER, ENCORI, and R projects. Expression of miRNAs was performed with R projects. Expression of lncRNAs was obtained from GEPIA. The association between H6PD and immune checkpoints in GBM was evaluated using GEPIA. Kaplan–Meier survival analysis and Cox regression were also utilized to assess the prognostic value of H6PD expression. A value of *p* < 0.05 was considered statistically significant in all analyses.

## 3. Results

### 3.1. Expression and Survival Analysis for H6PD in Pan-Cancer Studies

**Figure 1:**
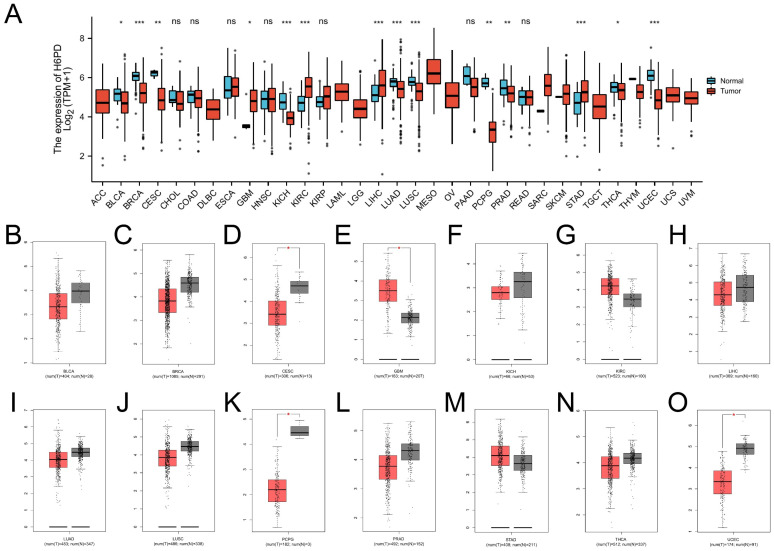
Expression analysis for H6PD in pan-cancer types**.** (**A**) The expression of H6PD in pan-cancer types of human cancer based on TCGA cancer and normal data. Differences in H6PD were detected in 14 cancers: bladder cancer (BLCA), breast cancer (BRCA), cervical squamous cell carcinoma (CESC), glioblastoma (GBM), kidney chromosome cancer (KICH), kidney renal clear cell carcinoma (KIRC), liver hepatocellular carcinoma (LIHC), lung adenocarcinoma (LUAD), lung squamous cell carcinoma(LUSC), pheochromocytoma and paraganglioma (PCPG), prostate adenocarcinoma (PRAD), stomach adenocarcinoma (STAD), thyroid cancer (THCA), and uterine corpus endometrial carcinoma (UCEC). (**B**–**O**) H6PD expression in 14 cancers was provided compared to corresponding TCGA and GTEx normal tissues. Differences in H6PD were detected in four cancers: CESC, GBM, PCPG, and UCEC. Briefly, H6PD was upregulated in GBM and downregulated in CESC, PCPG, and UCEC, suggesting that H6PD may play an oncogenic role in these four cancers. (ns: no significance; * *p*-value < 0.05; ** *p*-value < 0.01; *** *p* value < 0.001).

**Figure 2:**
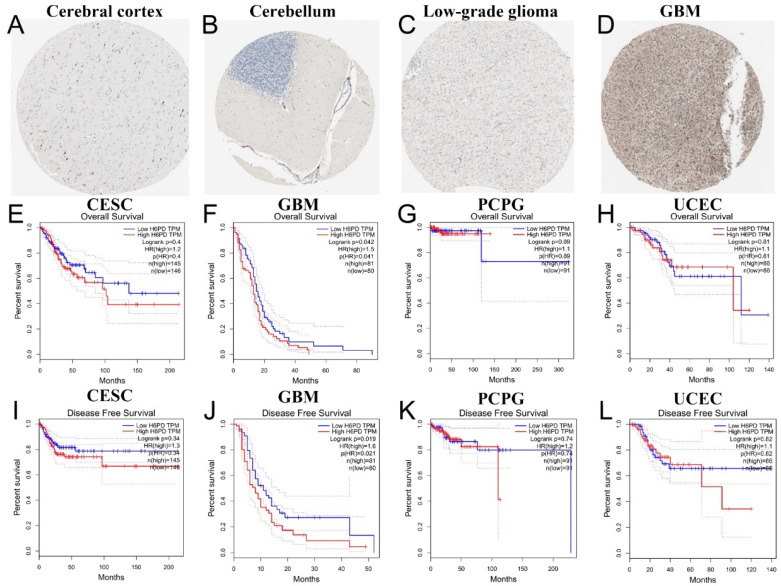
Immunohistochemistry and survival analysis of H6PD. (**A**–**D**) Low expression of H6PD was detected in the cerebral cortex, cerebellum, and low-grade glioma. Abundant H6PD expression was detected in GBM. (**E**–**L**) OS and DFS of CESC, GBM, PCPG, and UCEC are shown. For overall survival (OS) and disease-free survival (DFS), high expression of H6PD was significantly associated with poor prognosis in GBM patients. H6PD was not statistically significant in predicting the prognoses of patients with other cancer types.

Differences in H6PD were detected in 14 cancers based on TCGA cancer and standard data (Figure 1A): bladder cancer (BLCA), breast cancer (BRCA), cervical squamous cell carcinoma (CESC), GBM, kidney chromosome cancer (KICH), kidney renal clear cell carcinoma (KIRC), liver hepatocellular carcinoma (LIHC), lung adenocarcinoma (LUAD), lung squamous cell carcinoma (LUSC), pheochromocytoma and paraganglioma (PCPG), prostate adenocarcinoma (PRAD), stomach adenocarcinoma (STAD), thyroid cancer (THCA), and uterine corpus endometrial carcinoma (UCEC). H6PD expression in these 14 cancers was compared to corresponding TCGA and GTEx normal tissues (Figure 1B–O). Differential H6PD expression was detected in four cancers: CESC, GBM, PCPG, and UCEC. Briefly, H6PD was upregulated in GBM and downregulated in CESC, PCPG, and UCEC, suggesting that H6PD may play an oncogenic role in these four cancers. Low expression of H6PD was detected in the cerebral cortex, cerebellum, and low-grade glioma (Figure 2A–C). Abundant H6PD expression was detected in GBM (Figure 2D). For overall survival (OS) and disease-free survival (DFS), high expression of H6PD was significantly associated with poor prognosis in GBM patients (Figure 2E–L). H6PD was not statistically significant in predicting the prognoses of patients with other cancer types (Figure 2E–L).

### 3.2. Analysis of Candidate miRNAs and lncRNAs Binding to H6PD

**Figure 3:**
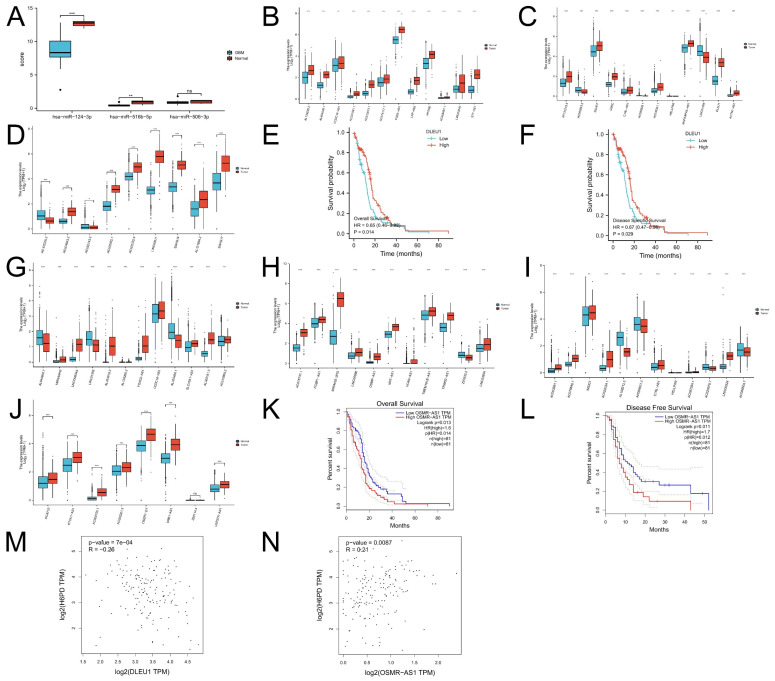
Expression, survival analysis, correlation of candidate miRNAs, lncRNAs, and H6PD. To ascertain whether some lncRNAs modulated H6PD, the upstream miRNAs bound to H6PD were predicted, and finally, three miRNAs, namely hsa-miR-124-3p, hsa-miR-516b-5p, and hsa-miR-506-3p, were found (**A**). Downregulation of hsa-miR-124-3p and hsa-miR-516b-5p was detected from GSE90603, while downregulation of hsa-miR-506-3p was not. Twenty-seven candidate lncRNAs related to hsa-miR-124-3p were upregulated in GBM (**B**–**D**). DLEU1 was significantly associated with prognosis in GBM patients, while the other 26 lncRNAs were not (**E**,**F**). OS and DFS analyses demonstrated that GBM patients with higher DLEU1 expression had a good clinical prognosis. Thirty-five candidate lncRNAs related to hsa-miR-516b-5p were upregulated in GBM (**G**–**J**). Statistical significance of OSMR-AS1 for predicting prognosis of GBM was detected, while statistical significance of the other 34 lncRNAs was not (**K**,**L**). GBM patients with higher OSMR-AS1 expression were negatively associated with OS and RFS. DLEU1 was negatively correlated with H6PD (**M**), while OSMR-AS1 was positively correlated with H6PD (**N**). (ns: no significance; * *p*-value < 0.05; ** *p*-value < 0.01; *** *p* value < 0.001).

According to the ceRNA hypothesis [13,14], there is a positive correlation between lncRNA and H6PD, while a negative correlation exists between miRNA and H6PD. We obtained three candidate miRNAs that could competitively bind to H6PD: hsa-miR-124-3p, hsa-miR-516b-5p, and hsa-miR-506-3p (Figure 3A). Downregulation of hsa-miR-124-3p and hsa-miR-516b-5p was detected from GSE90603, while downregulation of hsa-miR-506-3p was not. Subsequently, 27 candidate lncRNAs related to hsa-miR-124-3p were upregulated in GBM (Figure 3B–D). Statistical significance of DLEU1 for predicting the prognosis of GBM was detected, while statistical significance was not found for the other 26 lncRNAs (Figure 3E,F). OS and DFS analyses demonstrated that GBM patients with higher DLEU1 expression had a good clinical prognosis. Thirty-five candidate lncRNAs related to hsa-miR-516b-5p were upregulated in GBM (Figure 3G–J). Statistical significance of OSMR-AS1 for predicting the prognosis of GBM was detected, while statistical significance was not found for the other thirty-four lncRNAs (Figure 3K,L). GBM patients with higher OSMR-AS1 expression were negatively associated with OS and RFS. It was found that OSMR-AS1 was positively correlated with H6PD, while DLEU1 was negatively correlated with H6PD (Figure 3M,N). Considering expression analysis, survival analysis, and correlation analysis, OSMR-AS1 might be the highest-potential upstream lncRNA of the hsa-miR-516b-5p/H6PD axis in GBM.

### 3.3. Association of Immune Infiltration, Overall Survival, Immune Checkpoints, and H6PD Level in GBM

**Figure 4:**
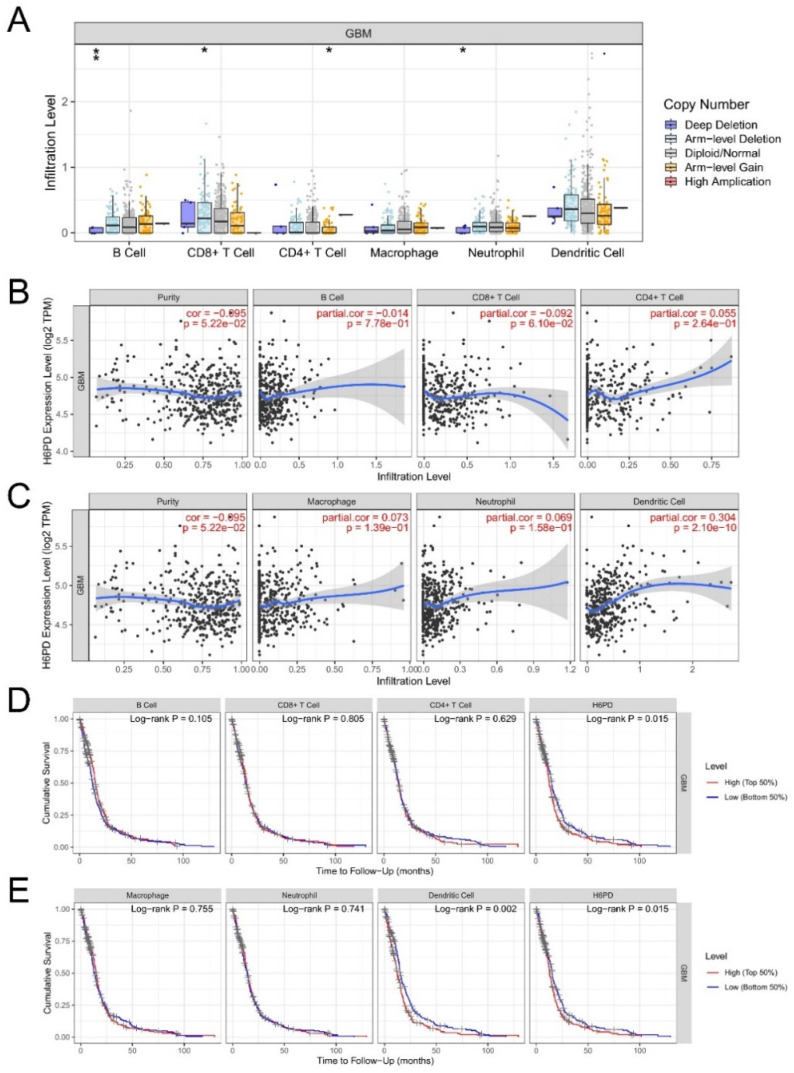
The relationship of immune cell infiltration and survival with H6PD levels in GBM. Infiltration levels of various immune cells under different copy numbers of H6PD in GBM were provided (**A**). A significant change in immune cell infiltration levels under various copy numbers of H6PD in GBM, including B cells, CD8+ T cells, CD4+ T cells, and neutrophils, was observed (**A**). The correlation of H6PD expression level with B cell, CD8+ T cell, CD4+ T cell, macrophage, neutrophil, and dendritic cell infiltration level in GBM was shown (**B**,**C**). H6PD expression was significantly positively associated with dendritic cells. In the survival module, we investigated whether immune infiltrates and H6PD expression were associated with clinical outcomes (**D**,**E**). Higher dendritic cell infiltration and expression of H6PD were significantly positively associated with poor outcomes. (* *p* < 0.05, ** *p* < 0.01).

**Figure 5:**
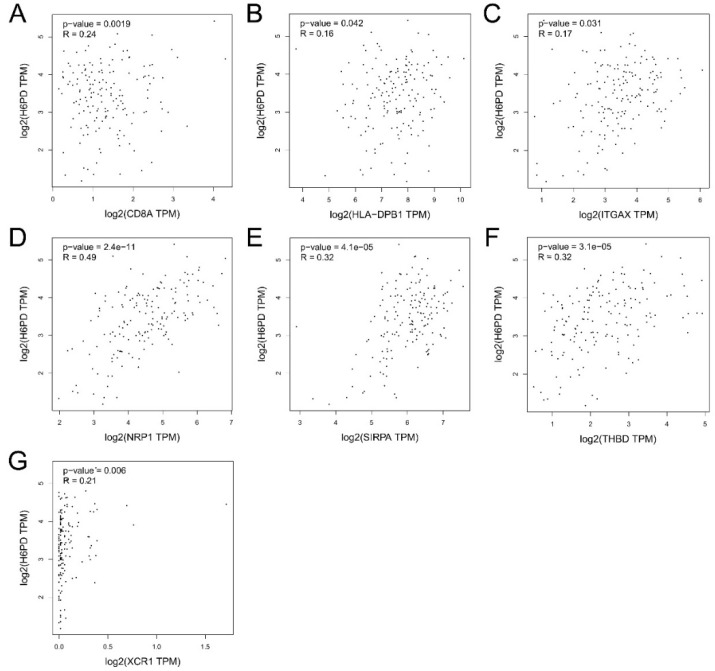
Correlation between biomarkers of dendritic cells and H6PD in GBM. CD8A, HLA-DPB1, TGAX, NRP1, SIRPA, THBD, and XCR1 were positively associated with H6PD expression (**A**–**G**).

**Figure 6:**
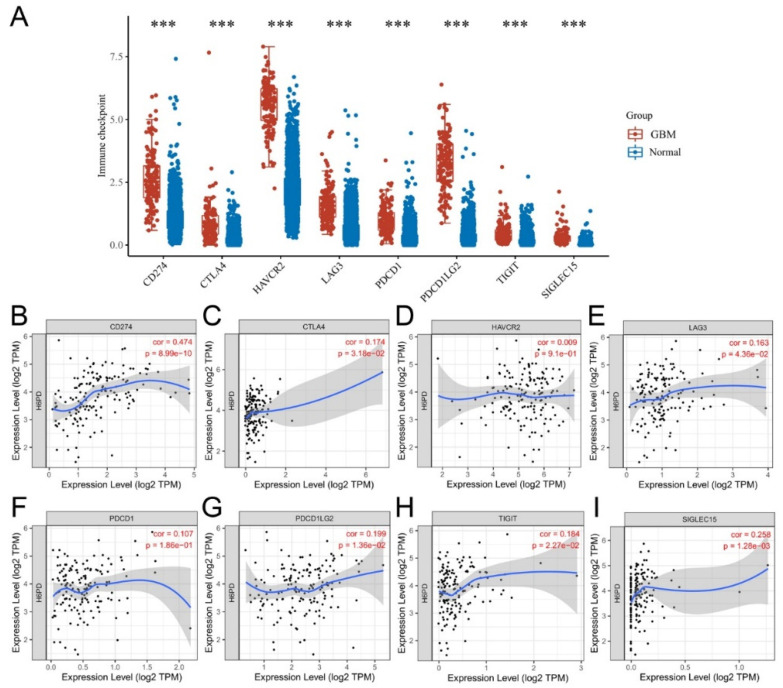
Relationship between H6PD and immune checkpoints in GBM. Higher expression of eight checkpoints in GBM was detected (**A**). Correlation between H6PD and immune checkpoints in GBM was assessed (**B**–**I**). H6PD expression was significantly positively correlated with CD274, CTLA4, LAG3, PDCD1LG2, TIGHT, and SIGLEX15. (***, *p* < 0.001).

Infiltration levels of various immune cells under different copy numbers of H6PD in GBM were provided. Significant changes in immune cell infiltration levels were observed under various copy numbers of H6PD in GBM, including B cells, CD8+ T cells, CD4+ T cells, and neutrophils. We found a significant positive correlation between H6PD expression and dendritic cell infiltration (Figure 4C). Higher dendritic cell infiltration and expression of H6PD were significantly positively associated with poor outcomes (Figure 4D,E). A correlation was found between biomarkers of dendritic cell infiltration and H6PD in GBM. CD8A, HLA-DPB1, TGAX, NRP1, SIRPA, THBD, and XCR1 were positively associated with H6PD expression (Figure 5 A-G). The association of H6PD with biomarkers of dendritic cells in GBM is summarized in Table 1. Higher expression of eight checkpoints in GBM was detected (Figure 6A). The correlations between H6PD and immune checkpoints in GBM were assessed (Figure 6B–I). H6PD expression was significantly positively correlated with CD274, CTLA4, LAG3, PDCD1LG2, TIGHT, and SIGLEX15.

## 4. Discussion

Malignant glioma is the most common primary brain tumor type and has a poor prognosis. According to previous studies, the median survival time for GBM patients after treatment is approximately one year [17]. Despite improvement in surgery and chemo-radiotherapy for GBM, the clinical prognosis of GBM patients is extremely poor. Accumulating evidence indicates that H6PD plays an essential role in tumor initiation, progression, and drug resistance in multiple human cancers, including gallbladder, prostate, and breast cancer [6,7]. However, the function, molecular mechanism, and immune infiltration of H6PD in GBM remain unclear.

The study conducted a pan-cancer analysis of H6PD’s expression using TCGA and GTEx data. H6PD was upregulated in GBM and downregulated in CESC, PCPG, and UCEC, suggesting that H6PD may play an oncogenic role in these four cancers. Although CECS, PCPG, and UCEC are different cancer types and come from different tissues, they share the same result from H6PD downregulation. H6PD controls cancer cell proliferation and migration through pleiotropic effects, unfolded protein response, calcium homeostasis, and redox balance, which may result in a downregulation in CECS, PCPG, and UCEC . Future studies assessing gene expression might provide necessary insight into the biological mechanism. Low H6PD expression was detected in normal brain tissues and low-grade glioma. Nevertheless, abundant H6PD expression was found in GBM and related to a poor prognosis. H6PD was not statistically significant in predicting the prognosis of patients with other cancer types. Previous studies also indicated that upregulation of H6PD plays an essential role in purine metabolic reprogramming and glioma stem cell progression [9]. 

To explore whether ncRNAs modulated H6PD, we predicted the candidate miRNAs of H6PD and finally found three miRNAs: hsa-miR-124-3p, hsa-miR-516b-5p, and hsa-miR-506-3p. Downregulation of hsa-miR-124-3p and hsa-miR-516b-5p was detected from GSE90603, while downregulation of hsa-miR-506-3p was not. Previous studies reported that miR-124-3p correlated with tumor proliferation and migration in glioma [18,19,20]. Hypoxia-induced PDIA3P1 promoted mesenchymal transition via sponging of miR-124-3p in glioma [18]. miR-124-3p inhibited the viability of glioblastoma by targeting RhoG [19]. miR-124-3p also prevented cell proliferation, migration, and invasion in glioma via downregulation of Fra-2 and EphA2 in glioma [20,21]. miR-516b-5p plays important roles in tumor initiation and progression of multiple human cancers, including hepatocellular carcinoma, retinoblastoma, bladder cancer, lung cancer, and esophageal squamous cell carcinoma [22,23,24,25,26]. Tumor-suppressive effects of LINC02362 were implemented by modulation of the miR-516b-5p in hepatocellular carcinoma [22]. miR-516b-5p binding with taurine-upregulated gene 1 led to ectopic H6PD expression in retinoblastoma [23]. 

The ceRNA hypothesis is a novel mechanism for RNA interactions. Based on the ceRNA hypothesis, lncRNA acts as a ceRNA to interact with mRNA by competing with the corresponding miRNA [13]. The ceRNA hypothesis has provided a novel perspective that ceRNA–miRNA–mRNA interactions (or lncRNAs acting as ceRNAs) can regulate many cancers’ biological processes for cancer therapy [27,28]. Thirty-five candidate lncRNAs related to hsa-miR-516b-5p were upregulated in GBM. It was found that OSMR-AS1 works as a prognosticator for GBM in the present study. GBM patients with higher expression of OSMR-AS1 possessed poor OS and RFS. A positive correlation between OSMR-AS1 and H6PD was detected. OSMR-AS1 was found to be a differentially expressed lncRNA and an independent prognosis factor for patients with hepatocellular carcinoma [29]. OSMR-AS1 was detected as a survival-related lncRNA in glioblastoma using TCGA databases [30]. Moreover, OSMR-AS1 was a lncRNA related to pro-neural to mesenchymal transition and was positively associated with poor prognosis in glioma [31].

Immune checkpoint blockading promotes immune infiltration and antitumor immune response [32]. Immune infiltration can contribute to tumor progression by altering the tumor microenvironment and extracellular matrix [14]. The relationship between H6PD and immune response remains unclear. Our work suggested that significant changes in immune infiltration with various copy numbers of H6PD, including B cells, CD8+ T cells, CD4+ T cells, and neutrophils, were observed in GBM. H6PD expression was significantly positively associated with dendritic cell infiltration. Moreover, higher immune cell infiltration of dendritic cells and higher expression of H6PD were significantly positively associated with poor outcomes. Subsequently, we found that biomarkers of dendritic cells, including CD8A, HLA-DPB1, TGAX, NRP1, SIRPA, THBD, and XCR1, were positively associated with H6PD expression. Additionally, we investigated the association between H6PD and immune checkpoints. Higher expression of eight checkpoints in GBM was detected (Figure 6A). The correlation between H6PD and immune checkpoints in GBM was assessed (Figure 6B–I). H6PD expression was significantly positively correlated with CD274, CTLA4, LAG3, PDCD1LG2, TIGHT, and SIGLEX15, indicating that targeting H6PD might increase the efficacy of immunotherapy in GBM. 

## 5. Conclusions

H6PD was highly expressed and correlated with an unfavorable prognosis in GBM. The OSMR-AS1/miR-516b-5p axis was identified as the highest-potential upstream ncRNA-related pathway of H6PD in GBM. Furthermore, the present findings demonstrated that H6PD blockading might possess antitumor roles via regulating dendritic cell infiltration and immune checkpoint expression. However, basic experimental studies and clinical trials need to be carried out in the future to further validate these bioinformatic analysis results for GBM.

## Figures and Tables

**Table 1:** Correlation between biomarkers of dendritic cells and H6PD in GBM.

DC Subtype	Biomarker	R-Value	*p* Value
cDC1	THBD	0.32	<0.001
	CLEC9A	-0.15	0.064
	CD8a	0.24	0.0019
	BATF3	0.0063	0.94
	XCR1	0.21	0.006
cDC2	CD1c	0.094	0.23
	CD172a/SIRPA	0.32	<0.001
pDC	CD303	0.031	0.7
	CD304/NRP1	0.49	<0.001
LC	CD1a	0.095	0.23
DC	HLA-DPB1	0.16	0.042
	HLA-DQB1	0.085	0.28
	HLA-DRA	0.013	0.87
	HLA-DPA1	0.13	0.091
	CD11c/ITGAX	0.17	0.031

## Data Availability

The datasets analyzed in this study are available in the following open access repositories. TGGA: The Cancer Genome Atlas (TCGA) database (https://tcga-data.nci.nih.gov/tcga/ (accessed on 19 April 2022)). GEPIA: http://gepia.cancer-pku.cn/detail.php (accessed on 19 April 2022). ENCORI: http://starbase.sysu.edu.cn (accessed on 19 April 2022). miRNet2.0: www.mirnet.ca/miRNet/home.xhtml (accessed on 19 April 2022). The UCSC Xena database: http://xena.ucsc.edu/ (accessed on 19 April 2022). TIMER: https://cistrome.shinyapps.io/timer/ (accessed on 19 April 2022).
